# 11β-Hydroxysteroid Dehydrogenase Type 1 Is Expressed in Neutrophils and Restrains an Inflammatory Response in Male Mice

**DOI:** 10.1210/en.2016-1118

**Published:** 2016-05-04

**Authors:** Agnes E. Coutinho, Tiina M. J. Kipari, Zhenguang Zhang, Cristina L. Esteves, Christopher D. Lucas, James S. Gilmour, Scott P. Webster, Brian R. Walker, Jeremy Hughes, John S. Savill, Jonathan R. Seckl, Adriano G. Rossi, Karen E. Chapman

**Affiliations:** Centre for Cardiovascular Science (A.E.C., T.M.J.K., Z.Z., C.L.E., J.S.G., S.P.W., B.R.W., J.R.S., K.E.C.) and Medical Research Council Centre for Inflammation Research (A.E.C., C.D.L., J.S.G., J.H., J.S.S., A.G.R.), Queen's Medical Research Institute, University of Edinburgh, Edinburgh EH16 4TJ, United Kingdom

## Abstract

Endogenous glucocorticoid action within cells is enhanced by prereceptor metabolism by 11β-hydroxysteroid dehydrogenase type 1 (11β-HSD1), which converts intrinsically inert cortisone and 11-dehydrocorticosterone into active cortisol and corticosterone, respectively. 11β-HSD1 is highly expressed in immune cells elicited to the mouse peritoneum during thioglycollate-induced peritonitis and is down-regulated as the inflammation resolves. During inflammation, 11β-HSD1-deficient mice show enhanced recruitment of inflammatory cells and delayed acquisition of macrophage phagocytic capacity. However, the key cells in which 11β-HSD1 exerts these effects remain unknown. Here we have identified neutrophils (CD11b^+^,Ly6G^+^,7/4^+^ cells) as the thioglycollate-recruited cells that most highly express 11β-HSD1 and show dynamic regulation of 11β-HSD1 in these cells during an inflammatory response. Flow cytometry showed high expression of 11β-HSD1 in peritoneal neutrophils early during inflammation, declining at later states. In contrast, expression in blood neutrophils continued to increase during inflammation. Ablation of monocytes/macrophages by treatment of CD11b-diphtheria-toxin receptor transgenic mice with diphtheria toxin prior to thioglycollate injection had no significant effect on 11β-HSD1 activity in peritoneal cells, consistent with neutrophils being the predominant 11β-HSD1 expressing cell type at this time. Similar to genetic deficiency in 11β-HSD1, acute inhibition of 11β-HSD1 activity during thioglycollate-induced peritonitis augmented inflammatory cell recruitment to the peritoneum. These data suggest that neutrophil 11β-HSD1 increases during inflammation to contribute to the restraining effect of glucocorticoids upon neutrophil-mediated inflammation. In human neutrophils, lipopolysaccharide activation increased 11β-HSD1 expression, suggesting the antiinflammatory effects of 11β-HSD1 in neutrophils may be conserved in humans.

Neutrophils are one of the first leukocytes recruited to an inflammatory site and are essential to fight microbial infections (1). They are short lived, surviving only a few hours in the circulation, and are released in huge numbers daily from the bone marrow as terminally differentiated cells. Neutrophils are recruited to sites of inflammation by microbial-derived products (eg, lipopolysaccharide [LPS]) and host-derived mediators such as cytokines (primarily IL-1β, IL-6, and TNF-α) and chemokines (eg, CXC chemokine ligand-8 and CXC chemokine ligand-5).

Endogenous glucocorticoids play a critical role in controlling inflammatory responses (2). Neutrophils, monocytes, and macrophages are all important targets for the antiinflammatory effects of glucocorticoids. The circadian rhythm in glucocorticoid release contributes to the normal circadian variation in inflammatory responses, including modulation of neutrophil recruitment (3). Furthermore, dysregulated hypothalamic-pituitary-adrenal axis activity is likely a contributory factor in chronic inflammatory conditions (4). Although neutrophils are important glucocorticoid targets, conflicting reports exist regarding their glucocorticoid sensitivity (5–7). Neutrophilic inflammation, as in some chronic lung diseases, is frequently relatively glucocorticoid resistant (8) for reasons that are unclear. Glucocorticoid sensitivity may differ between activated and nonstimulated neutrophils, and indeed, glucocorticoids delay neutrophil apoptosis (9), although not under severe hypoxia or in the presence of certain inflammatory mediators (10).

An important level of control over endogenous glucocorticoid action is exerted by the activity of 11β-hydroxysteroid dehydrogenase (11β-HSD), an enzyme that interconverts intrinsically inert glucocorticoids (cortisone, 11-dehydrocorticosterone) and their active forms (cortisol, corticosterone) (11). The type 2 isozyme, 11β-HSD2, which inactivates glucocorticoids, is not expressed in immune cells analyzed from healthy humans and mice (although it becomes expressed here in neoplasia and certain other pathological states [12–14]). However, the type 1 isozyme, 11β-HSD1 is widely expressed in immune cells in which it acts predominantly as an oxoreductase, increasing intracellular glucocorticoid levels. It can thus modulate and shape an ongoing immune or inflammatory response (reviewed in reference 15).

Normally in rodents, the levels of 11β-HSD1 substrate, 11-dehydrocorticosterone, are low in the circulation, but they are increased markedly when plasma corticosterone levels are elevated (16), for example, after the activation of the hypothalamic-pituitary-adrenal axis or corticosterone administration. Under conditions of chronic corticosterone excess, 11β-HSD1 becomes a major modifier of the ensuing adverse metabolic effects (17). 11β-HSD1 expression is frequently increased at sites of inflammation, including in humans, and is induced in fibroblasts and other cell types by proinflammatory cytokines, particularly IL-1β and TNF-α (15). Expression of 11β-HSD1 is very low in monocytes, but it is rapidly induced upon differentiation to macrophages, with macrophage activation being a powerful regulator of its expression (reviewed in reference 15). 11β-HSD1 expression has been reported in human neutrophils (18), although human neutrophils undergoing constitutive apoptosis are completely lacking in 11β-HSD1 oxoreductase activity (14).

We previously showed that 11β-HSD1 activity is high in inflammatory cells recruited to the peritoneum during thioglycollate-induced peritonitis in mice and is down-regulated as the inflammation resolves (19). During sterile peritonitis, 11β-HSD1-deficient mice show enhanced recruitment of inflammatory cells (20) and delayed acquisition of macrophage phagocytic capacity (19). However, the key cells in which 11β-HSD1 exerts these effects were unknown. Here we have identified activated neutrophils as the cells most highly expressing 11β-HSD1 during thioglycollate-induced peritonitis and show 11β-HSD1 expression in these cells is dynamically regulated during an inflammatory response. Furthermore, we have investigated the effects of acute inhibition of 11β-HSD1 upon neutrophilic inflammation during peritonitis.

## Materials and Methods

### Animals

To avoid interanimal variability that would be introduced due to differences in the stage of estrous in females, male mice were used. Male C57BL/6 mice (∼12–14 wk) bred on-site or purchased from Harlan were housed under controlled conditions (12 h light, 12 h dark cycle at 21°C) with unrestricted access to standard rodent chow and water. All experiments on animals were approved by the local ethics committee and were performed in accordance with the UK Home Office Animals (Scientific Procedures) Act of 1986.

### Thioglycollate-induced sterile peritonitis

Peritonitis was induced in mice by ip injection of 0.3 mL of 10% thioglycollate as described (19). Blood was collected from the tail vein into 3.9% sodium citrate. Peritoneal cells were collected by lavage with 5 mL cold PBS and counted using a hemocytometer. Bone marrow cells were collected by flushing femurs with 5 mL PBS. Blood and bone marrow cells were counted using a NucleoCassette and a NucleoCounter NC-100 (ChemoMetec). For the 11β-HSD1-inhibitor experiments, vehicle (saline with 2% dimethylsulfoxide) or compound UE2316 (10 mg/kg) (21) was administered by ip injection, the night before and 1 hour prior to thioglycollate injection. Peritoneal cells were collected 4 hours later.

### Isolation of mouse neutrophils

After lavage to collect peritoneal cells 24 hours after the thioglycollate injection, neutrophils were isolated by incubation with Ly6G-phycoerythrin antibody (BioLegend) followed by incubation with antiphycoerythrin secondary antibody magnetic beads (Miltenyi Biotec) according to the manufacturer's instructions. Generally, isolated peritoneal neutrophils were 96% or more pure, based on histochemical staining of cytocentrifuge preparations and flow cytometric analysis (Supplemental Figure 1).

### Isolation and treatment of human neutrophils

Neutrophils were isolated from the peripheral blood of healthy volunteers (Lothian Research Ethics Committee, 08/S1103/38) by dextran sedimentation and discontinuous Percoll gradient and resuspended in Iscove's modified Dulbecco's medium (PAA) with 10% autologous serum at 5 × 10^6^/mL (37°C, 5% CO_2_) as described (22). Neutrophil purity was routinely greater than 96% with 1%–3% contaminating eosinophils. Neutrophils (10^6^ cells) were seeded in RPMI 1640 medium containing 10% charcoal-stripped fetal bovine serum and were treated with 100 ng/mL LPS for 4 hours prior to RNA extraction and analysis.

### Flow cytometry

Cells from the peritoneum, bone marrow, and blood were incubated in PBS with 10% mouse serum (Sigma-Aldrich) for 20 minutes on ice to block nonspecific binding. Ly6G-phycoerythrin (PECy7), CD11b-PerCP Cy5.5, or Pacific Blue (BioLegend), 7/4-Alexa-647 (AbD Serotec) antimouse antibodies were added to the cell suspensions at concentrations recommend by the supplier and incubated on ice for 30 minutes in the dark. 11β-HSD1 sheep-derived antibody, generated in-house (23), was used in combination with donkey antisheep secondary antibody (Alexa Fluor 488; Invitrogen). Cells were treated with a fixation and permeabilization kit (Fix and Perm; Invitrogen) according to the manufacturer's instructions to allow for intracellular staining with the 11β-HSD1 antibody (Supplemental Figure 2). Blood and bone marrow cells were treated with BD lysis buffer (BD Biosciences) to eliminate red blood cells. Fluorescence was determined by FACScalibur using Cellquest (Becton Dickinson UK Ltd) or 5L LSR Fortessa using FACSDiva (Becton Dickinson UK Ltd) and analyzed using FlowJo software (Treestar).

### 11β-HSD1 activity assay

11β-HSD1 reductase activity in peritoneal immune cells was measured as described (19). Briefly, cells were incubated in medium containing 200 nM 11-dehydrocorticosterone with trace amounts of [^3^H]11-dehydrocorticosterone (made as described [19]). Steroids were extracted in triplicate at various time points and analyzed by HPLC as described (24).

### CD11b-diphtheria toxin ablation

Briefly, 24 hours prior to thioglycollate treatment, diphtheria toxin (25 ng/g body weight) was administered iv to CD11b-DTR transgenic mice to deplete monocytes/macrophages, as described (25). Quantification of cytocentrifuged cells collected 4 hours after thioglycollate injection showed a greater than 95% decrease in monocytes/macrophages in diphtheria toxin-treated CD11b-DTR transgenic mice. 11β-HSD1 reductase activity was measured in total peritoneal cells 4 hours after the thioglycollate injection.

### RNA extraction and real-time PCR analysis

Total mRNA was extracted from cells using Trizol (Invitrogen), and 1 μg mRNA was reverse transcribed using SuperScript III (Invitrogen). Specific mRNAs were quantified by real-time PCR (quantitative PCR) on a LightCycler 480 (Roche) as previously described (26, 27). The following primers (Invitrogen) and probes (Universal Probe Library; Roche) were used for PCR: *Hsd11b1*, probe 1, with forward, 5′-TCTACAAATGAAGAGTTCAGACCAG-3′ and reverse, 5′-GCCCCAGTGACAATCACTTT-3′; *Cd11b*, probe 16, with forward, 5′-AAGGATGCTGGGGAGGTC-3′ and reverse, 5′-GTCATAAGTGACAGTGCTCTGGA-3′; *Hprt*, probe 95, with forward, 5′-TCCTCCTCAGACCGCTTTT-3′ and reverse, 5′-CCTGGTTCATCATCGCTAATC-3′; *Sell*, probe 45, with forward, 5′-TGCAGAGAGACCCAGCAAG-3′ and reverse, 5′-CAGACCCACAGCTTCAGGAT-3′, and *Anxa1*, probe 95, with forward, 5′-GTGAACGTCTTCACCACAATTC-3′ and reverse, 5′-GTACTTTCCGTAATTCTGAAACACTCT-3′. For humans, the following primers were used: *HSD11B1* (Hs01547870_m1) and RPL32 (Hs00851655_g1) (Invitrogen).

### Statistics

Statistical analysis was performed using a Student's *t* test, a one-way ANOVA followed by Dunnett's, or a Tukey's multiple comparison test, as appropriate. Significance was set at *P* < .05. Unless stated otherwise, values are means ± SEM.

## Results

### The cells highly expressing 11β-HSD1 recruited to the peritoneum during peritonitis are neutrophils

**Figure 1.**
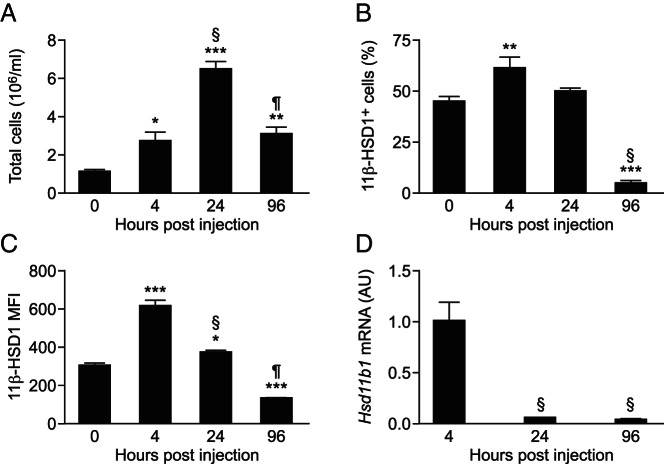
High 11β-HSD1 expression in inflammatory cells elicited to the peritoneum early during peritonitis. A, Peritoneal cell counts in lavages collected 0, 4, 24, and 96 hours after the injection of 300 μL of 10% thioglycollate. Flow cytometry was used to determine the number of 11β-HSD1^+^ peritoneal cells (as a percentage of total cells) (B) and the MFI of cellular 11β-HSD1 expression (C) during the course of peritonitis. D, Real-time PCR measurement of *Hsd11b1* mRNA levels (relative to *Hprt*) in total peritoneal cells 4, 24, and 96 hours after the injection of 300 μL of 10% thioglycollate, expressed in arbitrary units (AU), with levels at 4 hours arbitrarily set to 1.0. Data are means ± SEM and were analyzed by an ANOVA, with Tukey's post hoc tests. *, *P* < .05, **, *P* < .01, ***, *P* < .001, compared with 0 hours; §, *P* < .001 compared with 4 hours; ¶, *P* < .001 compared with 24 hours (n = 6–8/group).

After an ip injection of thioglycollate in C57BL/6 mice, the number of cells expressing 11β-HSD1 increased, as did the total peritoneal cell number. Both decreased as the inflammation resolved (Figure 1, A and B), consistent with previous measurements of 11β-HSD1 activity in thioglycollate-elicited peritoneal cells (19). In addition, compared with resident peritoneal cells (0 h), cellular expression of 11β-HSD1 (measured as mean fluorescence intensity [MFI]) was increased in recruited cells 4 and 24 hours after thioglycollate injection but was reduced as the inflammation resolved, with lower levels by 96 hours than in resident peritoneal cells (Figure 1C). The down-regulation of 11β-HSD1 MFI with resolution of inflammation at 96 hours may account for the reduction in 11β-HSD1^+^ cells at this time point (Figure 1B). Levels of the encoding *Hsd11b1* mRNA were high in the total peritoneal cells isolated 4 hours after thioglycollate injection, reducing dramatically by 24 hours (Figure 1D). These data confirm our previous findings of high 11β-HSD1 activity in cells recruited to the peritoneum during inflammation and also show the increase in activity is paralleled by high *Hsd11b1* mRNA levels, which subsequently decrease during the resolution phase.

**Figure 2.**
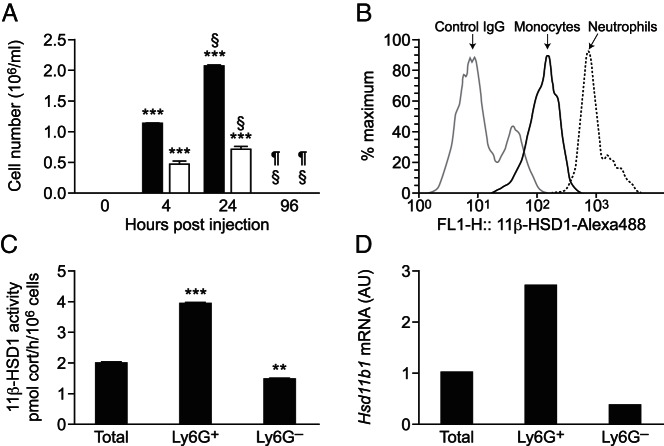
Thioglycollate-elicited neutrophils express 11β-HSD1. A, Flow cytometry was used to determine the number of neutrophils (black bars; Ly6G^+^7/4^+^CD11b^+^) and monocytes (white bars; Ly6G^−^7/4^+^CD11b^+^) in peritoneal lavages collected 0, 4, 24, or 96 hours after the injection of 300 μL of 10% thioglycollate. Data are means ± SEM and were analyzed by an ANOVA, with Tukey's post hoc tests, ***, *P* < .001, compared with 0 hours; §, *P* < .001 compared with 4hours; ¶, *P* < .001 compared with 24 hours (n = 6–8 mice/group). B, Representative histogram (of eight mice) from flow cytometry showing strong positive staining of 11β-HSD1 in neutrophils (black line), to a lesser degree in monocytes (dark gray line), and negative control staining (light gray) in cells lavaged 24 hours after the thioglycollate injection. C, 11β-HSD1 activity assay performed on freshly isolated neutrophils lavaged 24 hours after the thioglycollate injection: conversion of 200 nM [^3^H]-11-dehydrocorticosterone to corticosterone, expressed as picomoles corticosterone per hour per 10^6^ cells. Values are mean ± SEM of three independent pools of peritoneal cells (each from three mice) and were analyzed using an ANOVA with Tukey's post hoc tests. **, *P* < .01, ***, *P* < .001, compared with total cells. D, Real-time PCR measurement of 11β-HSD1 mRNA levels in purified neutrophils collected 24 hours after the thioglycollate injection. Each value represents a single pool of cells from five mice and is in arbitrary units (AU), with the level in total cells arbitrarily set to 1.0.

Both neutrophils (Ly6G^+^,7/4^+^,CD11b^+^) and monocytes (Ly6G^−^,7/4^+^,CD11b^+^) were detectable in the peritoneum 4 and 24 hours after thioglycollate injection, with neither population detectable in resident cells (0 h) or as inflammation was resolving (96 h after thioglycollate injection) (Figure 2A). Neutrophils and monocytes in the peritoneum stained positively for 11β-HSD1, with higher levels in neutrophils than in monocytes (Figure 2B). Separation of neutrophils from other peritoneal cells lavaged 24 hours after thioglycollate injection showed higher 11β-HSD1 reductase activity (conversion of 11-dehydrocorticosterone to corticosterone) and mRNA levels in isolated neutrophils than in total cells or the cells (mainly monocytes/macrophages), which remained after removal of neutrophils (Figure 2, C and D). Conversion of corticosterone to 11-dehydrocorticosterone was negligible in the peritoneal neutrophil population (3.3% conversion of 200 nM corticosterone to 11-dehydrocorticosterone by 10^6^ cells in 24 h), similar to previously reported background levels of dehydrogenase activity in total peritoneal cells (19).

**Figure 3.**
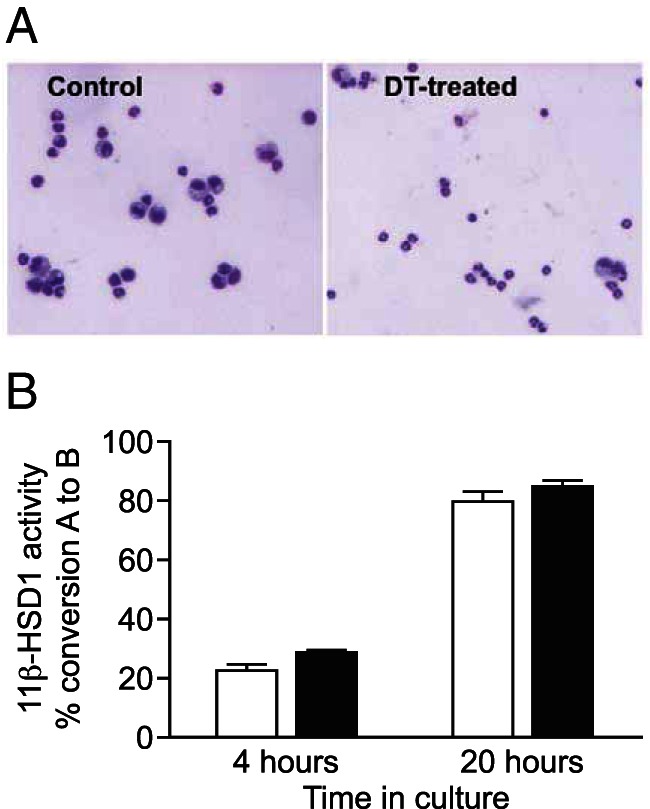
Monocyte depletion has no effect on the induction of 11β-HSD1 activity in thioglycollate-elicited peritoneal cells. Diphtheria toxin was administered to DTR transgenic mice 24 hours prior to the ip thioglycollate injection. Control mice were treated with thioglycollate alone. Peritoneal cells were lavaged 4 hours after the thioglycollate injection. A, May-Geimsa staining of cytocentrifuged peritoneal cells showing elicited neutrophils and monocytes from thioglycollate treated control mice (left panel), and depleted numbers of monocytes in lavages from diphtheria toxin (DT)-treated mice (right panel). B, 11β-HSD1 activity was measured as the percentage conversion of 200 nM [^3^H]-11-dehydrocorticosterone (11-DHC) to corticosterone in 10^6^ cells, measured after 4 and 20 hours of incubation with [^3^H]-11-DHC. White bars, controls; black bars, DTR transgenic mice. Photographs are representative of two different mice of each group, and values are mean ± range of conversion for two mice/group.

To confirm that neutrophils are the main population of cells expressing 11β-HSD1 during peritonitis, we used CD11b-DTR transgenic mice, in which the CD11b promoter drives the expression of the human diphtheria toxin receptor, to selectively ablate monocytes/macrophages (25). Injection of diphtheria toxin 24 hours prior to thioglycollate markedly depleted monocytes/macrophages (Figure 3) but had no significant effect on 11β-HSD1 activity in peritoneal cells lavaged 4 hours after the thioglycollate injection, consistent with neutrophils being the high 11β-HSD1-expressing cell type in the peritoneum at this time. Thus, although 11β-HSD1 is expressed in monocytes/macrophages, neutrophils account for most 11β-HSD1 activity in inflammatory cells recruited during sterile peritonitis.

### 11β-HSD1 expression in neutrophils is elevated by inflammation

**Figure 4.**
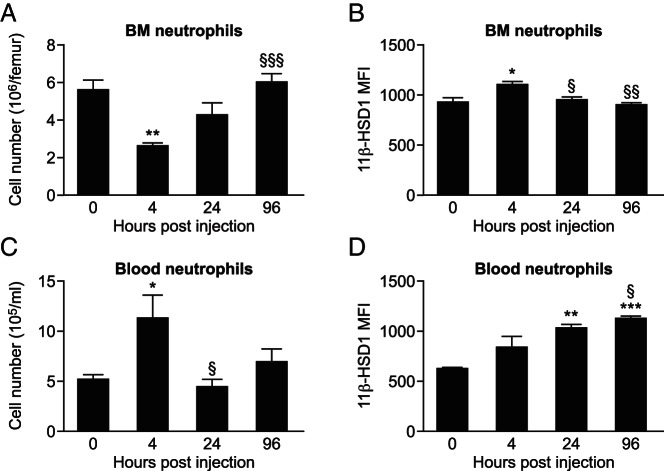
11β-HSD1 is up-regulated in neutrophils in blood and bone marrow during peritonitis. Neutrophil (Ly6G^+^7/4^+^CD11b^+^) cell number was determined by flow cytometry of freshly isolated (A) bone marrow (BM) and (C) blood from mice at various times after the thioglycollate injection. 11β-HSD1 MFI was measured in BM (B) and blood neutrophils (D) using flow cytometry. Data are means ± SEM and were analyzed by a one-way ANOVA followed by Tukey's post hoc tests. *, *P* < .05, **, *P* < .01, ***, *P* < .001, compared with 0 hours; §, *P* < .05, §§, *P* < .01, §§§, *P* < .001, compared with 4 hours (n = 6–8/time point).

We next asked whether inflammation per se increases 11β-HSD1 levels in neutrophils. Within 4 hours after the ip thioglycollate injection in mice, neutrophils were depleted in the bone marrow (Figure 4A), with an increased expression of 11β-HSD1 in those that remained (Figure 4B). The number of neutrophils in bone marrow and their expression of 11β-HSD1 returned to normal levels 24 hours after the thioglycollate injection (Figure 4, A and B). In contrast, the number of neutrophils in the blood was elevated 4 hours after thioglycollate injection, and despite normal blood neutrophil counts by 24 hours after the thioglycollate injection, 11β-HSD1 expression remained elevated in these cells (Figure 4, C and D). Similar to neutrophils, the number of monocytes in the bone marrow was reduced 4 hours after the thioglycollate injection and normalized within 24 hours (Supplemental Figure 3). In blood, total monocyte number was reduced 4 hours and 24 hours after the thioglycollate injection (Supplemental Figure 3). Moreover, 11β-HSD1 expression was increased in 7/4^hi^ and 7/4^med^ blood monocytes 24 hours after the thioglycollate injection (Supplemental Figure 3).

### Inhibition of 11β-HSD1 in vivo augments peritoneal cell infiltration and enhances CD11b surface expression on neutrophils during peritonitis

**Figure 5.**
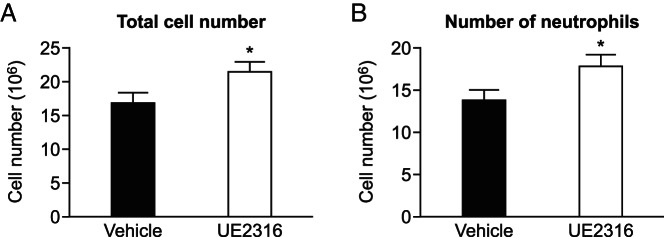
11β-HSD1 inhibition augments peritonitis. Lavages were collected from UE2316 pretreated mice and vehicle pretreated mice (black bars) 4 hours after the thioglycollate injection. Total peritoneal cell number (A) and the number of neutrophils (B) were greater in lavages of UE2316-pretreated mice (white bars) than in lavages from vehicle-pretreated mice (black bars). Data are means ± SEM and were analyzed by a Student's *t* test. *, *P* < .05 (n = 9–10).

To investigate whether 11β-HSD1 activity affects neutrophil recruitment during peritonitis, compound UE2316, a selective 11β-HSD1 inhibitor, was administered prior to the injection of thioglycollate. Addition of UE2316 to peritoneal cells in vitro confirmed the inhibition of 11β-HSD1 activity in these cells (Supplemental Figure 4). Injection of UE2316 alone did not elicit an inflammatory response, with no effect on peritoneal cell number, compared with vehicle (Veh) injected mice (peritoneal cell count: Veh, 1.9 ± 0.3 × 10^6^ vs UE2316, 2.0 ± 0.4 × 10^6^ cells/mL). Because there were no neutrophils or monocytes present in the peritoneum in the absence of inflammation, only experimental groups that received thioglycollate injection were compared. After the ip injection of thioglycollate, mice pretreated with UE2316 accumulated more inflammatory cells in the peritoneum (Figure 5A), including more neutrophils (Figure 5B), than mice that received Veh prior to thioglycollate injection. There was no significant effect of UE2316 on numbers of neutrophils in bone marrow or blood after the injection of thioglycollate (bone marrow: Veh, 2.64 ± 0.46 × 10^6^ cells per femur vs UE2316, 2.51 ± 0.54 × 10^6^ cells per femur (blood: Veh, 3.68 ± 0.76 × 10^6^ cells/mL vs UE2316, 2.95 ± 0.54 × 10^6^ cells/mL).

**Figure 6.**
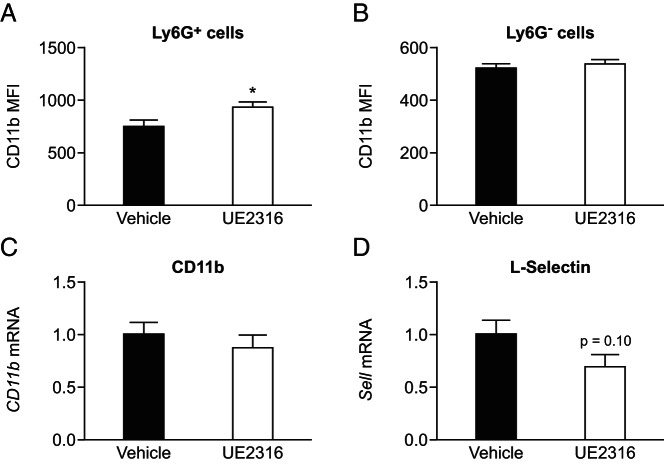
11β-HSD1 inhibition enhances surface expression of adhesion molecule CD11b on peritoneal neutrophils. Flow cytometry was used to measure surface expression of CD11b on peritoneal cells lavaged 4 hours after the thioglycollate injection. A, CD11b MFI was higher on the peritoneal neutrophils (7/4^+^Ly6G^+^) from mice pretreated with UE2316 (white bar) than from mice pretreated with vehicle (black bar). B, No difference was found in surface CD11b MFI on monocytes (7/4^+^Ly6G^−^) between the two groups. Data are means ± SEM and were analyzed by a Students *t* test. *, *P* < .05 (n = 9–10). Levels of mRNA encoding CD11b (C) and L-selectin (D) were measured relative to mRNA encoding HPRT in peritoneal cells lavaged 4 hours after the thioglycollate injection from mice pretreated with vehicle (Veh, black bars) or UE2316 (white bars), with levels in Veh-treated mice arbitrarily set to 1.0. Data are means ± SEM and were analyzed by a Student's *t* test. *, *P* < .05 (n = 8–9). HPRT, hypoxanthine-guanine phosphoribosyl transferase.

Cell surface expression of CD11b, an integrin family member that regulates leukocyte adhesion and migration, is a marker of neutrophil activation (28). By 4 hours after the thioglycollate injection, cell surface levels of CD11b were higher on the peritoneal neutrophils with prior inhibition of 11β-HSD1, compared with the mice pretreated with vehicle (Figure 6A), probably through increased CD11b mobilization to the cell surface rather than an increase in newly synthesized protein because *Cd11b* mRNA levels were not increased in peritoneal cells with inhibitor treatment (Figure 6C). Levels of mRNA encoding L-selectin, down-regulated on activated neutrophils, showed a trend (*P* = .10) to be lower in peritoneal cells with inhibitor treatment (Figure 6D), again suggesting greater neutrophil activation with 11β-HSD1 inhibition. There was no corresponding increase in cell surface CD11b expression on the 7/4^+^Ly6G^−^ monocyte population in the peritoneum 4 hours after the thioglycollate injection (Figure 6B) or on blood or bone marrow neutrophils (Supplemental Figure 5).

### Inhibition of 11β-HSD1 in vivo reduces gene expression of 11β-HSD1 itself in thioglycollate elicited peritoneal cells

**Figure 7.**
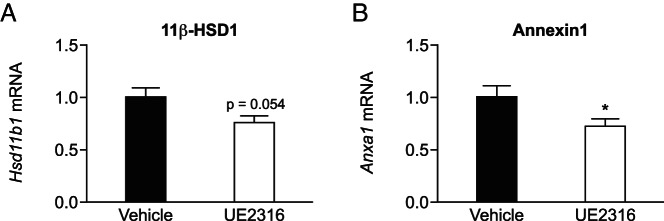
11β-HSD1 inhibition reduces the expression of 11β-HSD1 and annexin-1 during 4 hours of peritonitis. Quantitative PCR was performed on peritoneal cells collected 4 hours after thioglycollate injection of mice pretreated with vehicle (Veh, black bars) or UE2316 (white bars). Levels of mRNA encoding 11β-HSD1 (A) and annexin-I (B) were measured relative to mRNA encoding HPRT, with levels in Veh-treated mice arbitrarily set to 1.0. Data are means ± SEM and were analyzed using a Student's *t* test. *, *P* < .05 (n = 8–9). HPRT, hypoxanthine-guanine phosphoribosyl transferase.

Given the dynamic regulation of 11β-HSD1 in neutrophils during the inflammatory response, levels of the encoding mRNA were measured in peritoneal cells 4 hours after the thioglycollate injection, with pretreatment with inhibitor or vehicle. Pretreatment with the 11β-HSD1 inhibitor decreased levels of *Hsd11b1* mRNA (Figure 7A), suggesting autoregulation by glucocorticoids. Consistent with lower intracellular levels of the glucocorticoid, levels of *Anxa1* mRNA, encoding annexin-I, substantially expressed in neutrophils and glucocorticoid inducible (29), were also reduced in the thioglycollate-elicited peritoneal cells after the inhibitor treatment (Figure 7B).

### 11β-HSD1 is induced in human neutrophils by LPS

**Figure 8.**
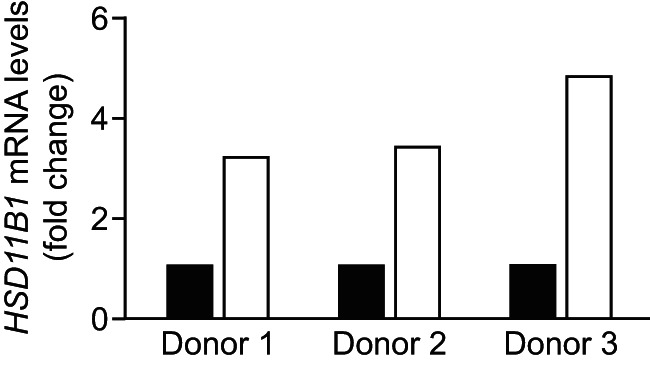
HSD11B1 mRNA is up-regulated in activated human neutrophils. Human neutrophils were isolated from blood of three healthy individuals and treated for 4 hours with 100 ng/mL LPS, prior to RNA extraction and real-time measurement of *HSD11B1* mRNA levels. Values are normalized to levels in untreated cells (black bars) and show fold induction of *HSD11B1* mRNA after LPS (white bars) for single individuals.

To investigate whether findings may extend to humans, we treated human neutrophils with LPS, an acute inflammatory stimulus, for 4 hours. *HSD11B1* mRNA, encoding 11β-HSD1 was induced in all three samples tested (Figure 8), suggesting that in humans as in mice, 11β-HSD1 is induced in activated neutrophils to restrain inflammation.

## Discussion

11β-HSD1 is expressed in myeloid cells (neutrophils, monocytes, macrophages, mast cells), but levels differ and depend on cellular activation state. Here we have identified neutrophils as the inflammatory cells that highly express 11β-HSD1 during sterile peritonitis. Inhibition of 11β-HSD1 increased early neutrophil-dominated inflammatory cell recruitment. This is similar to 11β-HSD1-deficient mice, which show increased inflammatory cell recruitment in sterile peritonitis and other models of neutrophil-driven inflammation, including carageenan-induced pleurisy, K/BxN serum-induced arthritis, and early after myocardial infarction (20, 30). These findings support an important role for 11β-HSD1 in neutrophils in restraining their accumulation at sites of sterile inflammation. Higher surface levels of CD11b and reduced expression of L-selectin suggest that 11β-HSD1 inhibition may reduce neutrophil rolling and increase neutrophil adherence within blood vessels, promoting greater extravasation of inflammatory cells. In addition to decreasing surface expression of CD11b (31), glucocorticoids suppress neutrophil adherence and emigration, in part through induction of annexin-1 (29). Consistent with the reduced intracellular glucocorticoid action, *Anxa1* expression in peritoneal cells was decreased with 11β-HSD1 inhibition, suggesting the impaired ability to increase annexin-1 levels is part of the mechanism that contributes to increased acute inflammation with 11β-HSD1 inhibition or deficiency.

As previously observed in total peritoneal cells and most other cell types, 11β-HSD1 activity was solely reductase with no dehydrogenase activity detected under the conditions used. In vivo inhibition of 11β-HSD1 reduced expression of the glucocorticoid-target gene, *Anxa1*, in inflammatory cells lavaged from the peritoneum early during inflammation when neutrophils predominate, consistent with reduced intracellular glucocorticoid reactivation. Our data are also consistent with *Hsd11b1* itself being a glucocorticoid target gene in neutrophils because mRNA levels were decreased with enzyme inhibition. *Hsd11b1* mRNA levels are autoregulated in many cells, with tissue levels in vivo increased under conditions of corticosterone excess (11, 17, 26). This feed-forward regulation of 11β-HSD1 suggests that when the hypothalamic-pituitary-adrenal axis is activated and substrate availability for 11β-HSD1 is increased (16), elevated levels of 11β-HSD1 in neutrophils will amplify glucocorticoid-mediated restraint of inflammation.

We observed a dissociation between levels of 11β-HSD1 protein (MFI) and the levels of the encoding mRNA, with protein expressed under conditions in which there is very little of the encoding mRNA. This has been commented on previously (32, 33) and could be due to a long protein half-life for 11β-HSD1 but a short half-life for the encoding mRNA. Our data support a long protein half-life. This situation would ensure that 11β-HSD1 protein is available to generate glucocorticoid if needed during an inflammatory response, even after plasma corticosterone and 11-dehydrocorticosterone levels return to normal. Reduced intracellular glucocorticoid regeneration (with reduced substrate levels) will remove the feed-forward system that maintains high levels of *Hsd11b1* mRNA under conditions of glucocorticoid excess.

The presence of *HSD11B1* mRNA has previously been reported in human neutrophils (18). Our data show it is up-regulated in human neutrophils in response to pathogenic stimulation, suggesting 11β-HSD1 may be important in regulating neutrophilic inflammation in humans. This highlights a possible side effect of 11β-HSD1 inhibition in humans. In clinical trials, selective 11β-HSD1 inhibitors are effective and well tolerated. Phase II clinical trials have shown a modest efficacy of selective 11β-HSD1 inhibitors in the improvement of glycemic control in patients with type 2 diabetes (reviewed in reference 34). Moreover, preclinical studies have suggested 11β-HSD1 inhibition may be beneficial in atherosclerosis and in age-related cognitive decline (34). Future clinical trials of 11β-HSD1 inhibition should be alert to situations in which neutrophilic inflammation is likely.

## Appendix

Antibody Table

**Table 1.** Antibody Table

Peptide/Protein Target	Antigen Sequence (if Known)	Name of Antibody	Manufacturer, Catalog Number, and/or Name of Individual Providing the Antibody	Species raised (Monoclonal or Polyclonal)	Dilution Used
Ly6G		Ly6G-phycoerythrin antibody	BioLegend, 127617		Neat, flow cytometry
CD11b		CD11b-PerCP Cy5.5	Biolegend 301327		Neat, flow cytometry
CD11b		CD11b-PerCP Pacific blue	Biolegend 101233		Neat, flow cytometry
Ly6B.2		7/4-Alexa-647	AbD Serotec, MCA771A647		Neat, flow cytometry
11β-HSD1	Recombinant mouse 11β-HSD1 protein	11β-HSD1	Generated in-house; De Sousa Peixoto et al (23)	Sheep, polyclonal	Neat, flow cytometry
